# Grain Size Effects on Mechanical Properties of Nanocrystalline Cu_6_Sn_5_ Investigated Using Molecular Dynamics Simulation

**DOI:** 10.3390/ma15113889

**Published:** 2022-05-30

**Authors:** Wei Huang, Kailin Pan, Bo Wang, Yubing Gong

**Affiliations:** 1 Engineering Research Center of Electronic Information Materials and Devices, Ministry of Education, Guilin University of Electronic Technology, Guilin 541004, China; huang0773@guet.edu.cn (W.H.); wangbo_guet@163.com (B.W.); gybcome@163.com (Y.G.); 2 School of Mechanical and Electrical Engineering, Guilin University of Electronic Technology, Guilin 541004, China

**Keywords:** nanocrystalline Cu_6_Sn_5_, grain size, mechanic properties, molecular dynamics simulation

## Abstract

Intermetallic compounds (IMCs) are inevitable byproducts during the soldering of electronics. Cu_6_Sn_5_ is one of the main components of IMCs, and its mechanical properties considerably influence the reliability of solder joints. In this study, the effects of grain size (8–20 nm) on the mechanical properties (Young’s modulus, yield stress, ultimate tensile strength (UTS), and strain rate sensitivity) of polycrystalline Cu_6_Sn_5_ were investigated using molecular dynamics simulations at 300 K and at a strain rate of 0.0001–10 ps^−1^. The results showed that at high strain rates, grain size only slightly influenced the mechanical properties. However, at low strain rates, Young’s modulus, yield stress, and UTS all increased with increasing grain size, which is the trend of an inverse Hall–Petch curve. This is largely attributed to the sliding and rotation of grain boundaries during the nanoscale stretching process, which weakens the interaction between grains. Strain rate sensitivity increased with a decrease in grain size.

## 1. Introduction

Sn-Ag-Cu (SAC) and Sn-Cu (SC) series solders are commonly used for soldering electronics [1,2,3]. At present, most electronic products involve copper pads; thus, the main compositions of intermetallic compounds (IMCs) produced in the soldering process are Cu_6_Sn_5_ and Cu_3_Sn [2,4]. IMCs are inevitable byproducts of the soldering process, and they considerably influence the mechanical and electrical bonding.

The thickness of IMCs is usually only a few microns, and it is challenging to measure their mechanical properties using conventional experiments. At present, the only alternatives are computer simulations. In terms of experiments: the hardness, Young’s moduli, and creep properties of IMCs have been studied by nano-indentation methods at different temperatures and loading rates [4,5,6,7,8,9]. In nano-indentation experiments, special samples need to be prepared, and due to the limited thickness of IMCs, it is difficult to obtain pure samples. Therefore, the reported hardness and Young’s moduli show considerable variations [6,8,10,11]. In computer simulations, two main methods are typically used to study the mechanical properties of IMCs, namely, first-principles calculations and molecular dynamics simulations. Due to the large number of computing resources required for first-principles calculations, the number of atoms able to be modelled is limited, and the mechanical properties of only single-crystal materials can be investigated [12,13,14,15,16]. Moreover, the influence of temperature cannot be considered in the calculation process, because of which the modelling assumes a temperature of 0 K. Molecular dynamics methods have greatly increased the number of atoms that can be calculated, even up to the micron scale [17,18,19,20,21,22,23].

A literature review showed that both experimental and computer models of IMCs suggest relationships between mechanical properties and temperature [6,18,20] as well as strain rate [8,18,20,24,25,26,27]. Studies have shown that grain size also affects the mechanical properties of materials [18,28,29,30,31,32,33,34,35,36]. There is some evidence that the mechanical properties of IMCs are also affected by grain size [18,35], but these studies are only based on single-crystal analysis. However, IMCs produced in the soldering process are polycrystals. It is necessary to study the effects of grain size on the mechanical properties of polycrystalline IMC.

In this study, the effects of grain size on the mechanical properties of polycrystalline Cu_6_Sn_5_ were studied by molecular dynamics simulations. The previous studies suggested that the mechanical properties of IMCs could be affected by strain rate [18,20,27]. Strain rate effects on polycrystalline Cu6Sn5 have been studied in our previous work [20], and they cannot be ignored. Therefore, in this study, the influence of grain size on the mechanical properties of IMCs with different strain rates was also studied.

## 2. Methodology

### 2.1. MEAM Potential

The molecular dynamics simulation in this study was carried out in a large-scale atomic/molecular massively parallel simulator (LAMMPS) [36], and the modified embedded atom method (MEAM) potential proposed by Baskes was selected [37]. According to the MEAM theory developed by Baskes et al., the total energy of the system ($E_{tot}$) comprises the energy embedding an atom in the background electron density and a pair interaction, as shown in Equation (1).

(1) $$E_{tot}=\sum_{i}\left[F_{i}\left({\bar{\rho }}_{i}\right)+\frac{1}{2}\sum_{j\left(\neq i\right)}\emptyset_{ij}\left(R_{ij}\right)\right]$$

where $F_{i}$ is the embedding energy of the atom *i*, $\emptyset_{ij}$ is the interaction potential between atoms *i* and *j*, and *R_ij_* is the distance between atoms *i* and *j.*

**Figure 1:**
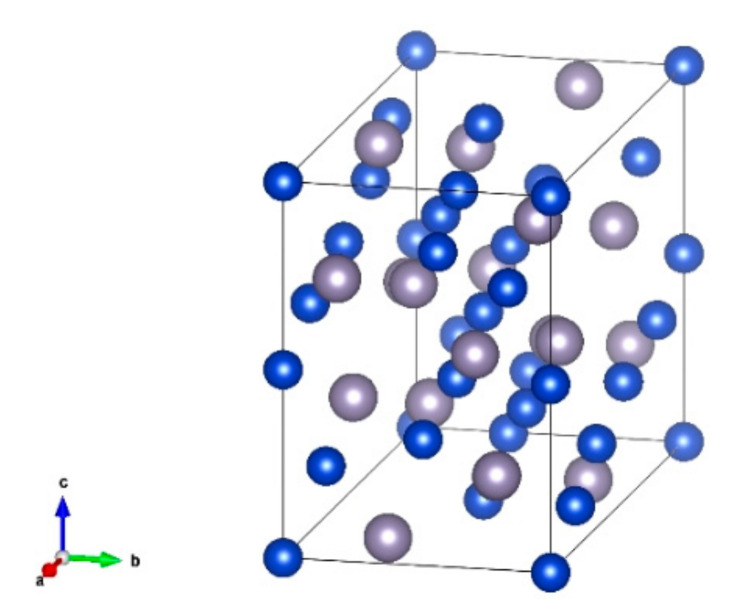
Cu_6_Sn_5_ (η’ phase) cell structure [16].

The Cu_6_Sn_5_ unit cell in this study is an η’ phase, which is a monoclinic crystal system with the C2/c space group [38], as shown in Figure 1 [16] (gray atoms represent Sn, and blue represents Cu). The parameters of the potential function selected refer to the literature values [20,39], and the validity of the potential functions has been verified in other studies [20].

### 2.2. Establishment of Polycrystals

**Figure 2:**
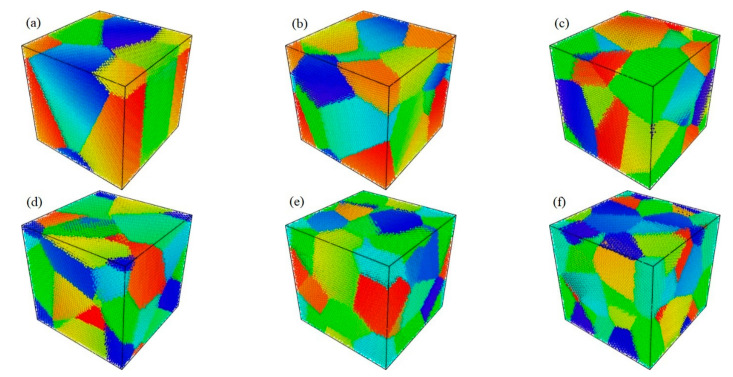
Polycrystalline Cu_6_Sn_5_ with average grain size of (**a**) 17 nm, (**b**) 14 nm, (**c**) 12 nm, (**d**) 11 nm, (**e**) 9 nm, and (**f**) 8 nm.

Polycrystals can be simulated using ‘Atomsk’ with the Voronoi tessellation rule [40]. In the process of polycrystalline establishment, box side length and seed number are chosen. In this study, a box with dimensions 200 Å × 200 Å × 200 Å was set, and the number of seeds varied from 2 to 30. Due to the constant volume of the box, the higher the number of seeds, the smaller the average size of the polycrystals. In this study, the average grain size was calculated by dividing the box size by the number of seeds. Then the average grain diameter was calculated, assuming a spherical volume to describe the grain. The grain size range in this study was, therefore 8–20 nm. Figure 2 shows polycrystals randomly generated when the numbers of seeds are 3, 6, 9, 12, 21, and 30, respectively. The corresponding average grain sizes in Figure 2a–f displayed using the Open Visualization Tool (OVITO) [41] are approximately 17, 14, 12, 11, 9, and 8 nm, respectively.

### 2.3. Simulation Settings

Periodic boundary conditions were set in all x, y, and z directions in the simulation, and the time step was set to 0.001 ps. First, the energy of the polycrystalline system was minimized, and then, the system was relaxed in the micro-canonical ensemble (NVE), canonical ensemble (NVT), and then the constant-pressure and constant-temperature ensemble (NPT) in turn. After the system stabilized, the polycrystals were stretched in an NPT ensemble. The ‘Fix deform’ command was adopted to allow the stretching of the polycrystals (at 300 K) with strain rates of 0.0001–10 ps^−1^.

## 3. Results and Discussion

**Figure 3:**
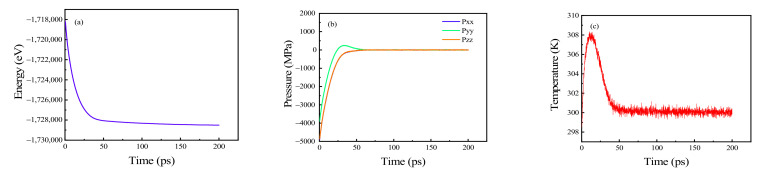
System states after relaxation of (**a**) total energy; (**b**) pressures in x, y, and z directions; and (**c**) temperature.

The polycrystals were allowed to relax in the NVE, NVT, and NPT ensembles with 1,000,000, 200,000, and 200,000 steps running, respectively, and then the system stabilized. Taking the polycrystal with four seeds as an example, the results of energy, pressure, and temperature after relaxation are shown in Figure 3. The total energy of the system stabilized at a minimum (Figure 3a); the pressure in the x, y, and z directions also stabilized at ~0 MPa (Figure 3b). In this study, polycrystals were stretched at 300 K, so the temperature of the final system was also stable near 300 K (Figure 3c).

### 3.1. Stress-Strain Responses at Different Strain Rates

**Figure 4:**
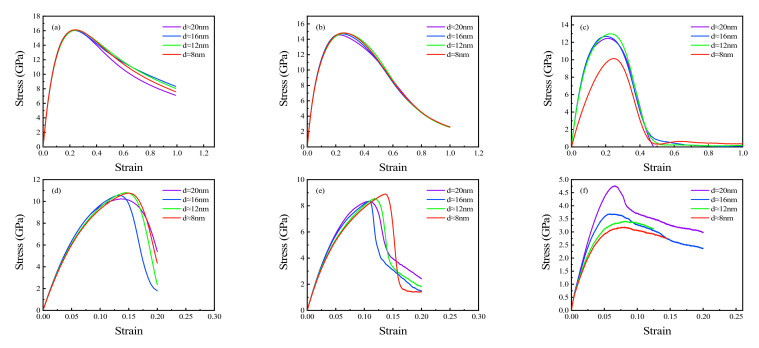
Stress-strain response of polycrystalline Cu_6_Sn_5_ at strain rates of (**a**) 10 ps^−1^, (**b**) 1 ps^−1^, (**c**) 0.1 ps^−1^, (**d**) 0.01 ps^−1^, (**e**) 0.001 ps^−1^, and (**f**) 0.0001 ps^−1^.

Polycrystalline Cu_6_Sn_5_ (average grain size = 8–20 nm) were stretched to study their stress–strain response at different strain rates (Figure 4a–f). The tensile strain rates selected in this study were 0.0001, 0.001, 0.01, 0.1, 1, and 10 ps^−1^, respectively. The variations in stress–strain curves with grain size were almost identical; thus, only the stress-strain curves with grain sizes of 8, 12, 16, and 20 nm were selected for further analysis. In the case of higher strain rates (10, 1, 0.1 ps^−1^; Figure 4a–c), the differences of stress-strain curves with different grain sizes were small. As the strain rate decreased (0.01, 0.001, and 0.0001 ps^−1^; Figure 4d–f), the differences in the stress–strain curves under different grain sizes became more obvious. These differences are discernible via the slope at the linear stage (Young’s modulus) and via yield stress and the UTS when stretching. The influences of grain size on Young’s modulus, yield stress, and UTS are analyzed in detail in the following sections. 

Studies have reported that when a polycrystal is stretched at a high strain rate, elastic deformation is dominant and brittle failure occurs; however, when stretched at a lower strain rate, the proportion of plastic deformation increases gradually before cracking [20,24,27]. Therefore, we concluded that grain size considerably influences plastic deformation under lower strain rate tension. This is attributed to the existence of grain boundaries, which make polycrystalline Cu_6_Sn_5_ more prone to dislocation during plastic deformation. When grain size changes, the proportion of grain boundary in the whole polycrystal will be different, which will eventually affect the degree of plastic deformation. Therefore, grain size has a greater influence on the stress–strain curve at a lower strain rate.

### 3.2. Elastic Properties of Polycrystalline Cu_6_Sn_5_

**Figure 5:**
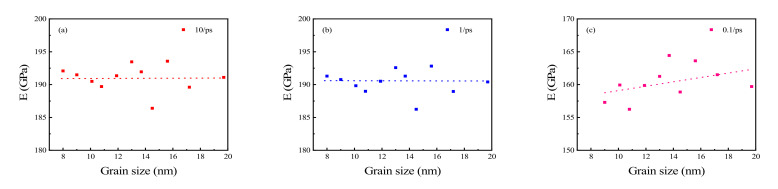

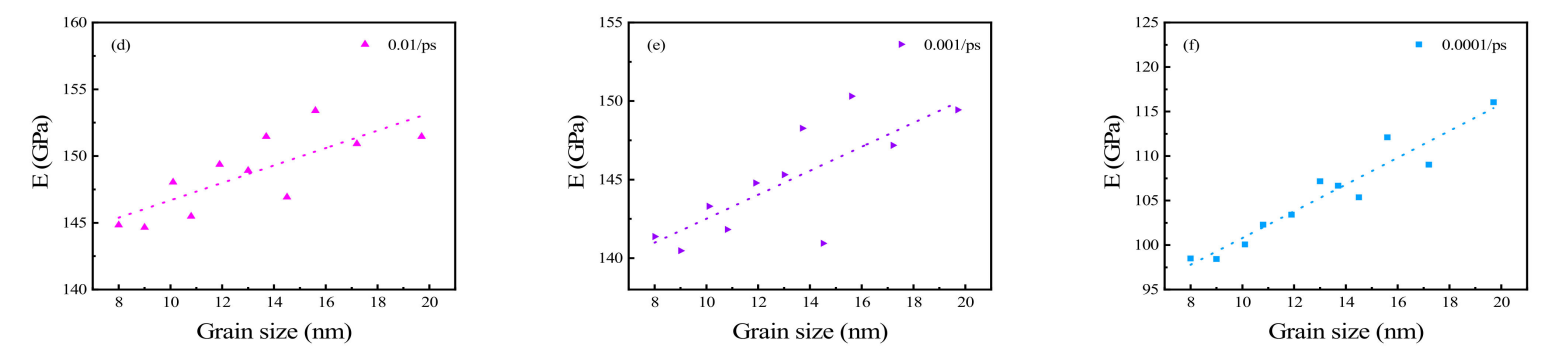
Young’s modulus of polycrystalline Cu_6_Sn_5_ at strain rates of (**a**) 10 ps^−1^, (**b**) 1 ps^−1^, (**c**) 0.1 ps^−1^, (**d**) 0.01 ps^−1^, (**e**) 0.001 ps^−1^, and (**f**) 0.0001 ps^−1^.

According to the generalized Hooke’s law, the relationship between stress and strain satisfies Equation (2), where *E* represents Young’s modulus, and *σ* and *ε* represent stress and strain, respectively, under linear deformation. In this study, the slope of the stress-strain curve was calculated in the strain range of 0–0.01 ps^−1^. The results are shown in Figure 5a–f, which are the Young’s moduli calculated at strain rates of 10, 1, 0.1, 0.01, 0.001, and 0.0001 ps^−1^, respectively.

(2) σ=E⋅ε

Young’s moduli at strain rates of 10 and 1 ps^−1^ fluctuated between 187 GPa and 193 GPa (Figure 5a,b). However, as the strain rate decreased, Young’s moduli showed an increasing trend with an increase in grain size (Figure 5c–f). In this study, the coefficient of variation (CV, the ratio of standard deviation to average value) was used to measure the dispersion degree of these Young’s moduli. The CVs of Young’s moduli are 0.01 at strain rates of both 10 and 1 ps^−1^. Therefore, it was concluded that when polycrystalline Cu_6_Sn_5_ was drawn at higher strain rates (10 and 1 ps^−1^ in this study), its Young’s modulus was almost unaffected by grain size, only fluctuating in a small range.

According to the iso-strain criterion, the Young’s modulus of a polycrystal can be expressed as Equation (3) [42], where *E_c_* represents the Young’s modulus of the polycrystal, $E_{1}$ and $E_{2}$ represent the Young’s modulus of grain and grain boundary, respectively, and $V_{1}$ and $V_{2}$ represent the volume fraction of grain and grain boundary, respectively. With an increase in grain size, the volume proportion of the grain boundaries in the whole polycrystal is larger. The grain boundaries comprise disordered atoms with weaker interactions between these atoms compared to the grain, so the Young’s modulus of the grain boundaries is lower than the Young’s modulus of the grain itself. Therefore, with the increase in grain size, the proportion of the grain boundaries in the polycrystal becomes smaller, and thus, the total Young’s modulus of the polycrystal increases.

(3) $$E_{c}=E_{1}V_{1}+E_{2}V_{2}$$

**Figure 6:**
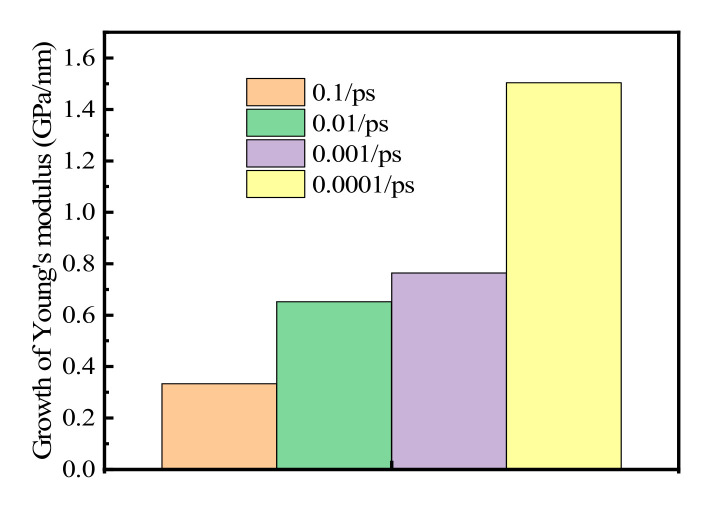
Growth rates of Young’s modulus with increasing grain size at varying strain rates.

The growth rate of Young’s modulus with grain size at strain rates of 0.0001–0.1 ps^−1^ is shown in Figure 6. The average Young’s modulus increased when the average grain size increased by 1 nm (Figure 5a–f: 0.33255, 0.65244, 0.76375, 1.50395 GPa at strain rates of 0.1, 0.01, 0.001, and 0.0001 ps^−1^, respectively). Therefore, Young’s modulus increases faster with increasing grain size at lower strain rates than that at higher strain rates. It was therefore concluded that the effect of grain size on Young’s modulus is greater at lower strain rates, which in turn means that it is more easily affected by grain size at a lower strain rate.

### 3.3. Yield Stress of Polycrystalline Cu_6_Sn_5_ with Different Grain Sizes

**Figure 7:**
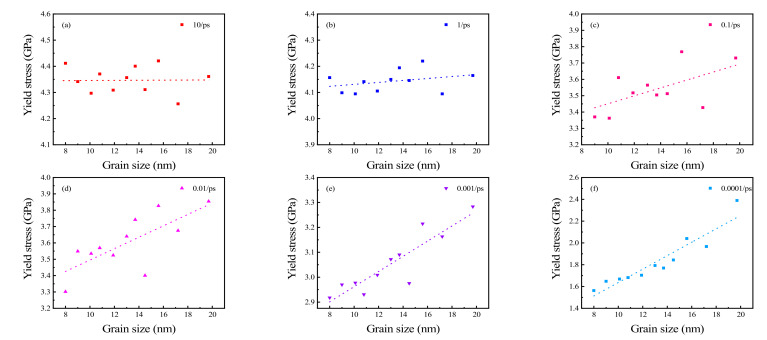
Yield stress of polycrystalline Cu_6_Sn_5_ at strain rates of (**a**) 10 ps^−1^, (**b**) 1 ps^−1^, (**c**) 0.1 ps^−1^, (**d**) 0.01 ps^−1^, (**e**) 0.001 ps^−1^, and (**f**) 0.0001 ps^−1^.

Since there is no obvious yield point during stretching, it is difficult to determine the maximum stress at which permanent deformation begins to occur. Thus, the 0.2% offset yield stress was used in this study. The results are shown in Figure 7. When the strain rate is 10 ps^−1^, the yield stress fluctuates randomly with changes in grain size. When the strain rate decreases from 1 to 0.0001 ps^−1^, the yield stress increases proportionally with an increase in grain size. As the strain rate decreases, the stability of this trend improves. The trends of yield stress with changing grain size at different strain rates closely mirror those of Young’s modulus.

### 3.4. Grain Size Effects on UTS of Polycrystalline Cu_6_Sn_5_

**Figure 8:**
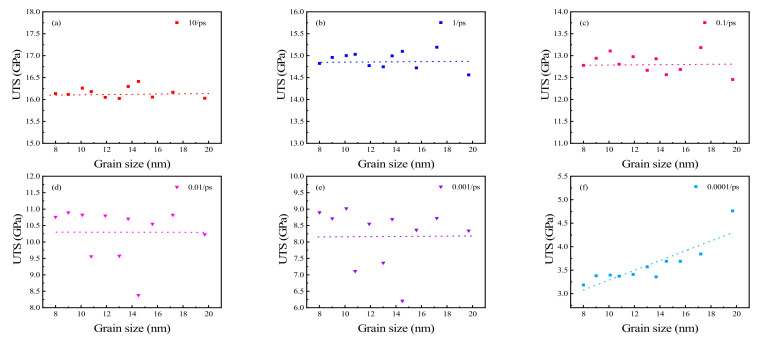
UTS of polycrystalline Cu_6_Sn_5_ at strain rates of (**a**) 10 ps^−1^, (**b**) 1 ps^−1^, (**c**) 0.1 ps^−1^, (**d**) 0.01 ps^−1^, (**e**) 0.001 ps^−1^, and (**f**) 0.0001 ps^−1^.

The effects of grain size on UTS at different strain rates were also analyzed. The results are shown in Figure 8. It was determined that UTS is almost unaffected by grain size when stretched at higher strain rates (10,1,0.1, 0.01, 0.001 ps^−1^: Figure 8a–e). The UTS fluctuated within the range of 16.0–16.5, 14.5 to 15.2, 12.4 to 13.2, 8.3 to 10.9, and 6.2 to 9.0 GPa with strain rates of 10,1,0.1, 0.01, and 0.001 ps^−1^, respectively. With a decrease in strain rate, the fluctuation range of UTS gradually increased. The CVs at the five strain rates mentioned above were found to be 0.008, 0.013, 0.018, 0.078, and 0.109, respectively. The corresponding mean UTS values are 16.16, 14.90, 12.83, 10.28, and 8.18 GPa, respectively. The UTS of the polycrystalline Cu_6_Sn_5_ with different grain sizes only fluctuated randomly within a very small range at rapid stretching (10 ps^−1^). As the strain rate decreased, the fluctuation range of UTS increased gradually (determined from the CV), but there was still no obvious trend. A likely explanation for this effect is plastic deformation increasing in polycrystals during the stretching process with a decrease in strain rate. Therefore, the plastic deformation which leads to a random fluctuation of UTS in a large range is not completely dominant (Figure 4a–e). At a strain rate of 0.0001 ps^−1^, the UTS increases with the increasing grain size, which conforms to the inverse Hall–Petch curve [28]. This is largely due to the plastic deformation of polycrystalline Cu_6_Sn_5_, which is dominant before it reaches UTS when stretched (Figure 5f). Grain boundary sliding and rotation, which weaken the nanocrystal [28,43,44], are the main mechanisms for the mechanical properties with grains < 30 nm [28].

### 3.5. Strain Rate Sensitivity of Polycrystalline Cu_6_Sn_5_

**Figure 9:**
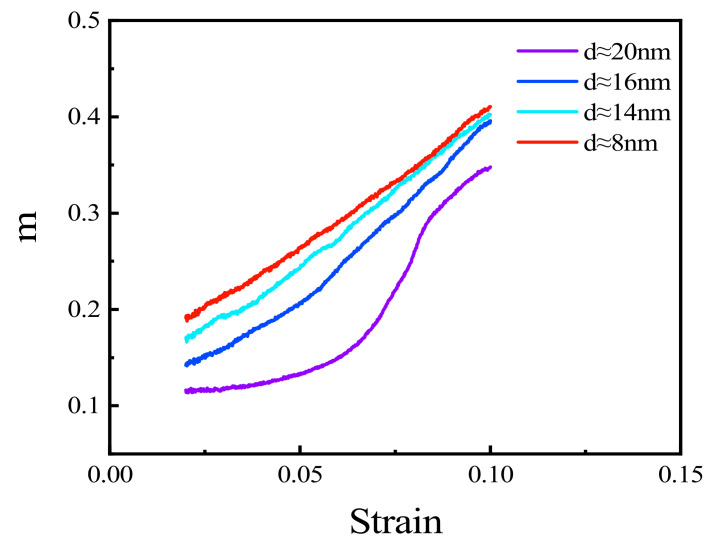
Strain rate sensitivity of polycrystalline Cu_6_Sn_5_ with varying grain sizes.

The above results demonstrate that the mechanical properties of polycrystalline Cu_6_Sn_5_ are affected by strain rate during tensile processes. Thus, strain rate sensitivity, *m*, is used to measure sensitivity, as shown in Equation (4) [45], where σ and $\dot{\varepsilon }$ represent flows stress and strain rate, respectively. Since the effects of grain size are more obvious at lower strain rates, the strain rates used in this calculation were 0.001 and 0.0001 ps^−1^, respectively. The strain range corresponding to flow stress is 0.02–0.08 (Figure 4e,f). Polycrystals with four grain sizes (20, 16, 14, and 8 nm) were selected for analysis. The results are shown in Figure 9. The results showed that the strain sensitivity of small grain size polycrystals is greater than that of larger grain size polycrystals. The strain rate sensitivity decreases with increasing grain size. At the same time, the strain rate sensitivity increases with increasing strain with the four-grain size polycrystals.

(4) $$m=\frac{\ln\left(\sigma_{2}/\sigma_{1}\right)}{\ln\left({\dot{\varepsilon }}_{2}/{\dot{\varepsilon }}_{1}\right)}$$

## 4. Conclusions

In this study, the effects of grain size on the mechanical properties of polycrystalline Cu_6_Sn_5_ were investigated. The results showed that the grain size affected both elastic and plastic deformation. Therefore, the grain size effects are different at different strain rates. The conclusions are as follows.

1. The effect of grain size on the stress-strain curve increases with decreasing strain rate and is practically invisible at high strain. This conclusion can be particularised for Young modulus, yield stress, and UTS.
2. Young’s modulus, yield stress, and UTS increase with the increasing grain size at a lower strain rate. Moreover, the growth rate of the Young’s modulus increases with a decrease in strain rate.
3. Polycrystals with a small grain size are more sensitive to strain rate than those with a large grain size. The strain rate sensitivity of polycrystalline Cu_6_Sn_5_ increases with increasing strain rate.

## Data Availability

All of the data supporting the reported results can be found in this manuscript.
